# Risk Factors that Affect Morbidity and Mortality in Patients with Perforated Peptic Ulcer Diseases in a Teaching Hospital

**DOI:** 10.4314/ejhs.v30i4.10

**Published:** 2020-07-01

**Authors:** Nebyou Seyoum, Daba Ethicha, Zelalem Assefa, Berhanu Nega

**Affiliations:** 1 Cardiothoracic Unit, Department of Surgery, School of Medicine, College of Health Sciences, Addis Ababa University, Ethiopia

**Keywords:** Perforation, Peptic ulcer, adverse outcome, Gastro-duodenal disease

## Abstract

**Background:**

This study was done to identify risk factors that affect the morbidity and mortality of patients operated for a perforated peptic ulcer in a resource-limited setting.

**Methods:**

A two years (January 1, 2016 -December 30, 2018) retrospective cross-sectional study was done on patients admitted and operated for PPU at Yekatit 12 Hospital, Addis Ababa, Ethiopia.

**Results:**

A total of 93 patients were operated. The median age affected was 29 years (Range 15–75 years). Male to female ratio was 7.5:1. Chewing chat, smoking and alcohol use were seen in 22 (23.6%), 35(37.6%), and 34(36.5%), cases respectively. Only 23.6% gave previous history of dyspepsia. The median duration of illness was 48hours and the duodenal to gastric ulcer perforation ratio was 6.5:1. In majority of the cases (63.3%) the perforation diameter was =10mm (63.3%). Cellan-Jones repair of the perforations was done in 92.5% of cases. A total of 47 complications were seen in 25 cases. The total complications and mortality rates were 25(26.8%) and 6(6.5%) respectively. The most common postoperative complication was pneumonia (13.97%) followed by superficial surgical site infection (10.8%). Mortality rate was highest among patients >50yrs [AOR (95%CI) =2.4(230)]. Delayed presentation of >24 hours [AOR (95%CI) =4.3(1.4–13.5)] and a SBP =90mmhg [AOR (95%CI) =4.8(1–24)] were found to be significantly related with higher complication rate.

**Conclusions:**

Patients who presented early and immediate corrective measures were instituted had better outcomes while those seen late developed unfavorable out-come with significantly higher complications. Therefore, early detection and treatment of PPU is essential.

## Introduction

The first clinical description of a perforated peptic ulcer (PPU) was made in 1670 in princess Henrietta of England (1). Since then, several notable people have succumbed to this illness over the years. PPU is a life-threatening complication occurring in about 2–14% of cases of peptic ulcer disease (2,3). Perforation is one of the commonest causes of emergency hospitalization (4–6). Its presentation may be dramatic with the pain of sudden onset often severe and radiating to the back with rapidly supervening features of peritonitis (7). Some studies have shown that patient factors like shock on arrival, acute renal failure, low serum albumin, metabolic acidosis and preoperative delay > 24 hours are significantly associated with a higher rate of mortality (8,9).

Globally, the incidence of PUD is declining, However, in developing countries like Ethiopia, despite the introduction of new drugs and recommended guidelines, PUD remains a substantial healthcare challenge (10–14). This study was undertaken to analyze the causes, presentation, and outcomes of surgical interventions done for PPU as measured by postoperative morbidity and mortality at Yekatit 12 Hospital Medical College.

## Methods And Materials

A retrospective cross-sectional study was done on patients admitted and operated on for PPU over three years (January 1, 2016 – December 30, 2018) at Yekatit 12 Hospital Medical College. The hospital is one of the tertiary hospitals located in Addis Ababa, which also receives patients from neighboring regions like Oromia, Amhara, and Southern Nations and Nationalities (SNNP).

The subject of this study included all patients aged fifteen and above, who underwent abdominal surgery for PPU. The hospital only accepts those aged ≥15 years for surgery. All patients were evaluated with history, physical examination, relevant laboratory and imaging studies. Following confirmation on the diagnosis, informed consent was obtained from all patients or their guardians before surgery. Patients were then put on intravenous fluids, nasogastric suction, and intravenous antibiotics. After adequate resuscitation, laparotomy was done through a midline incision and the perforation site identified. Simple closure of the perforation and reinforcement with a pedicle omental patch was done. Thorough peritoneal lavage with 4 to 5 liters of normal saline was done. The individual operating surgeon decides about the placement of an intraperitoneal drain. The operations were performed either by a senior resident or a consultant surgeon. All the patients were postoperatively put on double regime antibiotics consisting of Ceftriaxone (1gm bid) and Metronidazole (500 mg tid) and Omeprazole (20 mg bid). Patients were followed up subsequently for up to 6 months after surgery. Both morbidity and mortality during the hospital stay and the subsequent 6 months follow-up were included in the study.

Structured formats were used to collect relevant information. Data were collected from patients chart on dependent variable (morbidity and mortality) and the independent variables of the patients’ demographic data (age, sex), exposure risk (previous history of PUD, NSAID use, alcohol use, and cigarette smoking) and other clinical variables like duration of illness before surgical intervention, comorbid illness (Hypertension, Diabetes mellitus, HIV infection, Known cardiac and respiratory illness and others), deranged vital sign at presentation, site, and size of perforation, type of procedure done, use of drain and length of hospital stay.

Statistical analysis was done using SPSS version 23.0 software program. For continuous variables, the mean ± standard deviation, median, and ranges were calculated. Proportions and frequency tables were used to summarize categorical variables. The choice between parametric and non-parametric measures was made based on the distribution of the respective variables. While parametric measures were used for normally distributed variables, non-parametric tests were performed whenever the data was not normally distributed. All variables that were significant at the level of p-value = 0.20 in binary analysis were included in the multiple logistic regression model. The model was built with backward elimination to avoid multicollinearity. P-values less than 0.05 were considered statistically significant in the final model. Continuous predictor variables such as duration of illness were recoded and treated as categorical in the model.

### Deranged vital sign (V/S)

Patients presented with clinical evidence of either hypovolemic or septic shock with systolic blood pressure (SBP) =90mmhg.

### Patch failure

Leakage of gastrointestinal contents from a wound or drain, leaks are considered minor when small and resolve by their own and severe when surgical intervention is required.

The ethical approval for the study was obtained from the research and publication office of the Department of Surgery at Addis Ababa University.

## Results

### Socio-demographic characteristics

**Figure 1:**
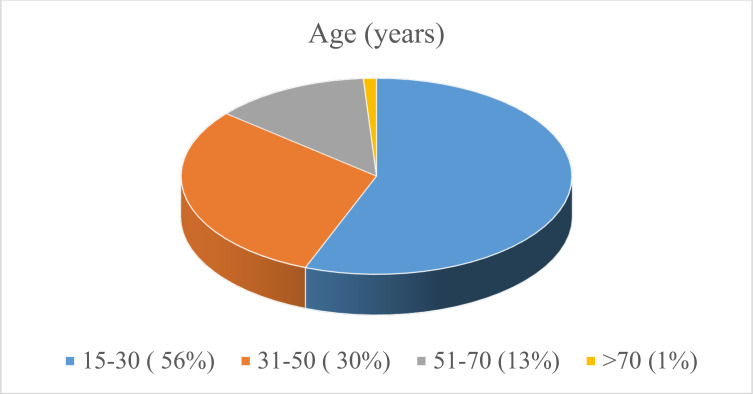
A pie-chart that shows the age distribution of Perforated PUD patients at Yekatit 12 hospital medical college from January 1, 2016 – December 30, 2018

Out of 93 patients included in this study, there were 82 (88.2%) male and 11 (11.8%) female patients (M: F →7.5:1). The median age of presentation was 29 years with an interquartile range of 16 and the majority of patients affected; [52(55.9)] were between 15–30 years (Figure 1)

Most of the patients, 85(91.3%) had formal education either primary school or above, and more than three-quarters of them were employed. Half of the patients [45(48.4%)] were married and were Orthodox [62 (66.7%)] in religion.

### Clinical presentation and investigations

The median average duration of symptoms before arrival was 48 hours with an interquartile range of 72 hours. Patients who were referred from other healthcare facilities arrived after an average of 22.5 hours later than otherwise. Only 28(30%) of the patients reached the hospital in 24 hours of the onset of symptoms. The commonest symptoms seen were sudden onset of diffuse abdominal pain [87 (93.5%)], nausea/vomiting [80(86%)], abdominal distension [50(53.4%)], constipation [20 (21.5%)], diarrhea [8 (8.6%)], and fever [5 (5.3%)]. Right lower quadrant pain that mimics symptoms of appendicitis was seen in 3(3.2%) patients. Abdominal tenderness and signs of peritonitis were demonstrated in 87(93%) of them, 11(11.8%) had presented with deranged V/S. Seventy-one (76.3%) patients reported neither symptoms nor treatment of previous PUD. Those with previous history, [22 (23.6%)], had symptoms and occasional treatment for a duration ranging from 1 – 14 years. Even though chewing Khat is a common practice in Ethiopia, it is only 22(23.6%) patients reported routine use. Other risk factors like smoking, regular alcohol consumption and regular NSAIDs use were reported in 35(37.6%), 34(36.5%) and 9(9.7%) patients respectively. Most patients who smoke also consume alcohol regularly.

In this study, 17(18.3%) patients had associated comorbidity; namely, hypertension, Diabetes mellitus, COPD, HIV, pulmonary tuberculosis, major depression, alcohol dependency, malnutrition and other medical illnesses (Table 1).

**Table 1:** Descriptive statistics on the sociodemographic and clinical presentation of perforated PUD patients at Yekatit 12 Hospital Medical College from January 1, 2016 – December 30, 2018

Variables	Number (%)
Age in year	
15–30	52(55.9)
31–50	28(30.1)
51–70	12(12.9)
>70	1(1.1)
Sex	
Male	82(88.2)
Female	11(11.8)
Place of residence	11(11.8)
Urban	85(91.4)
Rural	8(8.6)
Clinical Presentation	
Severe abdominal pain	87(93.5)
Vomiting	80(86)
Abdominal distension	50(53.4)
Constipation	20(21.5)
Diarrhea	8(8.6)
Fever	5(5.3)
Right lower quadrant pain	6(6.5)
Generalized peritonitis	85(91.4)
Epigastric tenderness	5(5.4)
Tachycardia	52(55.9)
Deranged Vital sign	11(11.8)
Comorbidity	
Hypertension	2(2.2)
Diabetes Mellitus	1(1.1)
HIV infection	1(1.1)
COPD	1(1.1)
Others	12(12.9)
Total	17(18.3)

The mean hemoglobin value was 15 ± 2.4 mg/dl, and no anemia was detected. Fifty-two (56%) patients had leukocytosis. Albumin was determined for 18(19.3%) and a mean value of 2.9 ± 1.2 mg/dl was found. H. pylori test was done in 19 patients of whom 6(31.6%) were positive. Seventy-one (76.3%) of the study participants had plain abdominal and chest radiographs that demonstrated air under the diaphragm.

### Operative findings

After arrival at the hospital, patients were operated on within an average of 5 ± 3.6 hours. During surgery, the expulsion of gas was demonstrated in all cases. An average of 600ml with IQR of 1200ml of GI content was sucked out from the general peritoneum. Most perforations were found in the anterior duodenum [78(83.9)], whereas 12(12.9%) had perforation at the antral part of the stomach. The duodenal to gastric ulcer perforation ratio was 6.5:1. All patients had single perforation, and no recurrence was identified. One patient developed perforation after a stoma ulcer after gastrojejunostomy.

Three (3.2%) cases had minor perforation (=5mm) that was sealed naturally with omentum. For those patients with sealed perforations, peritoneal lavage with warm saline alone was done. One of those three patients subsequently developed intra-abdominal sepsis and died of MOF. Looking into the size of perforations, 57(63.3%) were of minimal size (≤ 10mm) and 33(35.5%) were massive (>10 mm). The peritoneal fluid was bilious in 21(22.6%), purulent in 21(22.6%) and mixed in 48(51.6%) patients. Histological examination of tissue taken from the stomach was done for seven patients and it revealed no malignancy.

Almost all operating surgeons, 91(97.8%), were resident trainees. The majority of the operations, 60(65.2%), were done during duty time. Pedicle omentum patching (Cellan Johns procedure) was done in 86(92.5%) patients, whereas patching and biopsy were done in 5(5.4%) patients. During operation, there was one iatrogenic rectal injury for whom de-functional colostomy done. A sub-hepatic drain was left universally, 89(95.7%), in almost all cases.

The median length of hospital stay was 8 days (ranging from 5–50 days). Upon discharge of the patients, only 76.3% of patients received H. pylori eradication treatment.

### Postoperative complications (Morbidity)

A total of 47 complications were seen in 25(26.8%) patients. The leading early complications were pneumonia, 13(13.9%), followed by surgical wound infection 10(10.8%), intra-abdominal abscess 8(8.6%), patch failure 6(6.5%), ARF 6(6.5%), postoperative ileus 4(4.3%), complete wound dehiscence 3(3.2%), and entero-cutaneous fistula, 1(1.1%). Among those who developed postoperative complications, 8(8.6%) patients underwent re-laparotomies for intra-abdominal abscess collection with patch failure, [6(6.4%)], and complete wound dehiscence, [3(3.2%0], iatrogenic rectal injury, [1(1.1%0[ and abscess collection without patch failure [1(1.1%)], one patient underwent re-laparotomy 3 times. A total of 6(6.5%) patients died during the study period. The causes of death were a failure of the patch with subsequently generalized peritonitis in five patients and an initial intractable septic shock with subsequently MOF in one patient.

Late complications that require re-admission were seen in four (4.3%) patients. The reasons for readmissions were small bowel obstruction, complicated pneumonia, gastric outlet obstruction, and incisional hernia.

### Factors predicting morbidity

In the logistic regression models, the duration of illness, deranged V/S, and the size of perforation was identified to have a significant association with morbidity (Complications). Those patients who presented after > 24 hours of illness were 5x more likely to develop complication [COR (95%CI) =5.1 (1.8–13.7)], and those patients who presented with low deranged V/S had 6x more likely to develop complication [COR (95%CI) =6.2(1.6-23.6)]. Similarly, patients with larger perforation (10–30mm) were 3.5x more likely to develop a complication.

**Table 2:** Descriptive statistics on the type and frequency of complications encountered following surgery for perforated PUD patients at Yekatit 12 hospital medical college from January 1, 2016 -December 30, 2018.

Variables	Number (%)
Pneumonia	13(13.9)
Wound infection	10(10.8)
Intra-abdominal abscess	8(8.6)
ARF	6(6.5)
Patch failure	6(6.5)
Wound Dehiscence	3(3.2)
Postoperative ileus	4(4.3)
Entero-cutaneous fistula	1(1.1)
Other complications*	20(21.5)
Death	6(6.5)

* UTI, DVT, Anemia, Malnutrition, DIC, Atelectasis, Septic shock

After adjusting for confounding factors, the duration of illness and shock were found to be the only significant predictors of complications. Patients who presented after >24 hours of illness were 4x more likely to develop complications [AOR (95%CI) = 4.3(1.4–13.5)], and patients who presented with deranged V/S were 5X more likely to develop complication [AOR (95%CI) = 4.8(1–24)] (Table 3).

**Table 3:** Factors that were associated with the morbidity of Perforated PUD patients at Yekatit 12 Hospital Medical College from January 1, 2016 - December 30, 2018

Predictors		Morbidity		COR	95%CI		P-value	AOR	95%CI		P-value
		Yes	No								
Age	≥50 years	6	6	3.26	0.94	11.3	0.062	2.8	0.6	12	0.17
	>50 years	19	62	1	-	-	-	1	-	-	-
Comorbidity	Yes	7	10	2.26	0.75	6.78	-	-	-	-	-
	No	18	58	1	-	-	-	-	-	-	-
Sex	Male	20	62	0.39	0.1	1.4	0.12	-	-	-	-
	Female	5	6	1	-	-	-	-	-	-	-
Duration of illness	= 24 hours	7	45	1	-	-	-	1	-	-	-
	> 24 hours	18	23	5.1	1.8	13.7	0.002	4.3	1.4	13.5	0.01
Systolic BP	≥90mmhg	18	64	1	-	-	-	1	-	-	-
	=90mmhg	7	4	6.2	1.6	23.6	0.007	4.8	1	24	0.05
Pulse rate	≥100bpm	14	38	1.01	0.34	2.5	0.99	-	-	-	-
	=100bpm	11	30	1	-	-	-	-	-	-	-
Site of perforation	Duodenal	18	60	1	-	-	-	1	-	-	-
	Stomach	7	8	2.9	0.9	9	0.07	2.1	0.55	8.2	0.3
Size of perforation	=10mm	10	47	1	-	-	-	1	-	-	-
	10 to 30mm	14	19	3.5	1.3	9.2	0.01	1.4	0.4	5	0.5
Type of fluid sucked out	Bilious	8	16	1	-	-	-	-	-	-	-
	Purulent	4	17	0.5	0.12	2	0.3	-	-	-	-
	Mixed	13	35	0.7	0.3	2	0.5	-	-	-	-
Time of operation	Day	11	21	1	-	-	-	-	-	-	-
	Night	14	46	0.6	0.3	1.5	0.3	-	-	-	-

### Factors predicting mortality

The logistic regression model showed that deranged V/S and age were identified to be significantly associated with mortality. Age ≥50 years had 20x the risk of dying compared to age less than fifty years [COR (95%CI) = 19.6(3–125)]. Patients who presented with deranged V/S were 10x more likely to die compared to those who presented without it [COR (95%CI = 9.8(1.7–57)]. After adjustment for confounding factors, age was found to be the only significant predictor of mortality. Patients who were ≥50 years had 2.4 times chance of dying compared to = 50 years [AOR (95%CI) = 2.4(0.230)] (Table 4).

**Table 4:** Factors that were associated with mortality of Perforated PUD patients at Yekatit 12 Hospital Medical College from January 1, 2016 -December 30, 2018.

Predictors		Mortality	COR	95%CI	P-value	AOR	95%CI	P-value
		Yes	No								
Age	≥50 years	4	8	19.6	3	125	0.002	2.4	2	30	0.005
	=50 years	2	79	1				1	-	-	-
Comorbidity	Yes	2	15	2.4	0.4	14	0.34	-	-	-	-
	No	4	72	1	-	-	-	-	-	-	-
Sex	Male	4	78	4.3	0.7	27	0.1	-	-	-	-
	Female	2	9	1	-	-	-	-	-	-	-
Duration of	≤ 24 hours	1	51	1	-	-	-	1	-	-	-
illness	> 24 hours	5	36	7	0.8	63	0.08	0.5	0.02	14	0.7
Systolic BP	≥90mmhg	3	79	1	-	-	-	1	-	-	-
	<90mmhg	3	8	9.8	1.7	57	0.01	0.43	0.03	6	0.5
Pulse rate	≥100bpm	2	50	2.7	0.5	15	0.27	-	-	-	-
	<100bpm	4	37	1?	-	-	-	-	-	-	-
Site of	Duodenum	4	74	1	-	-	-	-	-	-	-
perforation	Stomach	2	13	0.4	0.1	2	0.3	-	-	-	-
Size of	<10mm	1	56	1	-	-	-	1	-	-	-
perforation	10-30mm	4	29	0.13	0.1	1.2	0.07	0.2	0.005	5	0.3
Type of fluid	GI content	1	23	1	-	-	-	-	-	-	-
sucked out	Pus	2	19	0.4	0.03	5	0.5	-	-	-	-
	Pus and GI content	3	45	0.6	0.06	6.6	0.7	-	-	-	-
Time of	Day	2	30	1	-	-	-	-	-	-	-
operation	Night	4	56	0.9	0.16	5.4	0.9	-	-	-	-
Complication	Yes	6	19	-	-	-	-	-	-	-	-
	No	0	68	-	-	-	-	-	-	-	-

## Discussion

This study demonstrated that PPU is a universal emergency surgical problem that predominately affects young males with low incidence of previous symptoms or treatment for PUD. Similar to other studies done in Ethiopia (10,11) and regional countries (12–16), the majority of our patients presented late after 24 hours of initial symptoms, and such delays and presence of preoperative deranged V/S were found to be significant predictors of complications. Age ≥ 50 years was also found to be significantly associated with higher mortality.

During the three years’ study period, a total of 103 patients were operated on with an average of 34 cases annually. This finding is consistent with similar studies done in Ethiopia (10,11) while it was much bigger than what was reported by Moses et al (12) from Liberia, Ugochukwu et al from Nigeria (13), Phillipo et al (14) from Tanzania, Schein et al (17) and Mieny et al (18) from South Africa. These differences may reflect differences in the rate of risk factors for PPU or a difference in the catchment population size of the respective hospitals. To understand this phenomenon better, further study is required.

Males predominated in our study (M: F ®7.5:1), and this finding is also reflected in other studies from developing countries where the male to female ratio range from 1.3: 1 to 9:1. (13–19). Reports from developed countries often showed that incidence is higher in elderly females taking ulcerogenic medications (20,21). In our series, only 9.7% had a history of ingestion of NSAIDs. The higher incidence of PPU amongst young males in our community could be attributed to excessive use of Chat (*Katha Edulis*), smoking, and alcohol. It is known that smoking inhibits pancreatic bicarbonate secretions, which tend to neutralize acid secretion, thus predisposing to increased acidity in the duodenal bulb. It also causes a delay in duodenal ulcer healing (22). Alcohol, on the other hand, predisposes to gastric ulceration, stimulates gastric acid secretion as well as enhancing gastrin release (23). Chronic ingestion of Chat predisposes to gastritis and duodenitis (24); however, its cause-effect relationship in PPU has not been so far established.

More than two-thirds of our patients presented after 24 hours of acute symptom onset. This could be because of the lack of patients’ awareness, poor transportation systems, and failure to detect and refer patients early. Patients referred from other healthcare facilities arrived after an average of 22.5 hours late than otherwise. Such a delay also resulted in higher morbidity (Table 3). Similarly, Svanes C. (25) reported that adverse outcomes increased markedly when the delay exceeds 12 hours. According to him, delay of more than 24 hours increased lethality seven-to eight-fold, complication rate three-to four-fold and length of hospital stay two-fold, compared to a delay of 6 hours or less.

Similar to other studies, establishing the diagnosis of PPU has primarily relied on plain radiographs of the abdomen/chest that was demonstrated in 76.7% of cases (23–26). Literature showed that 80-90% of cases of PPU could be diagnosed with simple erect abdominal or chest x-ray. During difficulty, CT-scan taken with oral contrast study is considered as a gold standard that can even detect small pneumoperitoneum (38). Abdominal ultrasonography has also been found to be superior to plain radiographs in the diagnosis of free intraperitoneal air (37). In our study, none of these other imaging studies were used for the diagnosis of PPU.

In our study, a duodenal to gastric ulcer ratio was found to be 6.5:1. A higher ratio was reported in Kenya (11.5:1), Tanzania (12.7:1) and Sudan (25:1); (14,27,28). Lower ratio of 3:1 to

4:1 was reported from developed countries (27,29). In Ghana, even a higher incidence of gastric perforation was reported (29). Gastric ulcer is considered a rare disease in Africa being 6–30 times less common than duodenal ulcers (28,29,30). Looking into the influence of the perforation site on adverse outcome, some reports showed a higher mortality rate with PPU of gastric origin, which is not demonstrated in our study (31,32,33).

Several surgical techniques could be used to treat PPU. Primary closure by interrupted sutures, closure by interrupted sutures covered with a pedicle omentum on top of the repair (Cellan-Jones repair) and plugging the perforation with a free omental plug (Graham patch) are the most common techniques used to treat PPU. In this study, Cellan-Jones repair was done in 86 (92.5%) patients. A similar procedure of choice was reported in another series too (35). During laparotomy, the findings usually vary according to the duration, site and sizes of perforation. Unlike studies done by Dodiyi-Manuel A et al (19) and Nuhu et al (39) where 88.9% and 82.7% of perforations were large size, we encountered large perforation in only 35.5% of cases. Some studies demonstrated that morbidity and mortality were significantly increased when the perforation diameter was wider than 0.5 cm in PPU (34). In this study, the perforation diameter did not influence morbidity.

In our study, sub-hepatic drain was left almost universally in all cases (95.7%). However, there is no evidence to support that leaving a drain reduces the incidence of intra-abdominal collections. On the contrary, it may lead to infection of the drain site and increase the risk of intestinal obstruction. (5).

Our finding of a total of 47 complications happened in 25 cases of the study participants, making the complication rate 27%. This finding is very similar to the with study done by Soro Kountele Gona et al from Côte d’Ivoire (36) and Phillipo et al from Tanzania (16) with 27.5% and 29.8% complications respectively. However, it is low as compared to report from KIMS Hospital, from India with 72.1% (37) and Nigeria (13) with a 63.2 % complication rate. Six patients developed patch failure of which five died with a case fatality rate of 83.33%. As compared to other studies done by Kin Tong Schein (5) where their leak rate is 2.1%, our result (6.5%) was much higher.

Without test for H. pylori, 76.3% of the study participants were given eradication therapy upon discharge while 23.7% were not given and are prone to develop recurrence. Current evidence showed that omeprazole and triple therapy treatment after simple closure of PPU reduces the incidence of recurrence ulcer rates significantly (40). Therefore, we recommend that intravenous proton pump inhibitor be given for 72–96 hours after surgery, start oral triple therapy when immediately after oral intake is initiated. In order to establish *H. pylori* eradication, urea breath test should be done after completion of the medical treatment.

Finally, even though this study was the first of its type to be conducted in Yekatit 12 Hospital, it also has some limitations. Since it is a single institution experience, it may not adequately reflect the whole population. Failure to adequately document the postoperative complications may have underestimated it. Besides, since the duration of postoperative follow-up was short, we could not estimate the long-term effect of surgery.

In conclusion, PPU remains a frequent clinical problem in our society predominantly affecting young males not known to have previous PUD. The higher incidence of risk factors like regular consumption of chat, alcohol, and cigarette among patients with PPU could be related to it. To minimize its incidence, proper public health education should be given, especially to vulnerable age groups. In our hospital, despite patients’ late presentation, in the majority of cases, simple closure of PPU with omental patch followed by Helicobacter pylori eradication treatment was found to be effective with excellent results. However, a corrective measure to minimize patch failure that was responsible for the majority of deaths should be done.
